# Hydrogen‐Doped *c*‐BN as a Promising Path to High‐Temperature Superconductivity Above 120 K at Ambient Pressure

**DOI:** 10.1002/advs.202408275

**Published:** 2024-10-07

**Authors:** Han‐Bin Ding, Rui Niu, Shen‐Ao Li, Ying‐Ming Liu, Xiao‐Jia Chen, Hai‐Qing Lin, Guo‐Hua Zhong

**Affiliations:** ^1^ Shenzhen Institute of Advanced Technology Chinese Academy of Sciences Shenzhen 518055 China; ^2^ Nano Science and Technology Institute University of Science and Technology of China Suzhou 215123 China; ^3^ University of Chinese Academy of Sciences Beijing 100049 China; ^4^ Department of Physics and Texas Center for Superconductivity University of Houston Houston TX 77204 USA; ^5^ School of Science Harbin Institute of Technology Shenzhen 518055 China; ^6^ School of Physics Zhejiang University Hangzhou 310058 China

**Keywords:** c‐BN, electron–phonon coupling, first‐principles calculations, superconductivity, superhard

## Abstract

Finding high‐temperature superconductivity in light‐weight element containing compounds at atmosphere pressure is currently a research hotspot but has not been reached yet. Here it is proposed that hard or superhard materials can be promising candidates to possess the desirable high‐temperature superconductivity. By studying the electronic structures and superconducting properties of H and Li doped *c*‐BN within the framework of the first‐principles, it is demonstrated that the doped *c*‐BN are indeed good superconductors at ambient pressure after undergoing the phase transition from the insulating to metallic behavior, though holding different nature of metallization. Li doped *c*‐BN is predicted to exhibit the superconducting transition temperature of ≈58 K, while H doped *c*‐BN has stronger electron–phonon interaction and possesses a higher transition temperature of 122 K. These results and findings thus point out a new direction for exploring the ambient‐pressure higher‐temperature superconductivity in hard or superhard materials.

## Graphical abstract

By doping H atom into c‐BN, the superconducting transition temperature above 120 K are predicted to be achieved in superhard material at ambient pressure, which is far higher than those in graphite, fullerene, and other carbides and nitrides. These results and findings thus point out a new direction for exploring the ambient‐pressure higher‐temperature superconductivity in hard or superhard materials.

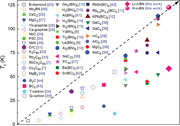

## 1 Introduction

High‐temperature superconductivity is one of the hottest topics in the physics community in 2023, reflecting people's desire for room‐temperature superconductors at ambient pressure. At present, the recognized ambient‐pressure high‐temperature superconductors with the transition temperature (*T*_*c*_) exceeding the boiling point temperature of liquid nitrogen (77 K) are copper oxides, the maximum *T*_*c*_ reaches 135 K in Hg–Ba–Ca–Cu–O system at ambient pressure.^[^ ^1^ ^]^ The *T*_*c*_ values of the highly concerned hydride superconductors exceed those of copper oxide compounds, reaching a level close to room‐temperature, such as lanthanum hydride,^[^ ^2^, ^3^ ^]^ yttrium hydrogen,^[^ ^4^, ^5^ ^]^ and lanthanum‐yttrium ternary hydrides.^[^ ^6^ ^]^ However, the near room‐temperature superconductivity of hydrides requires a pressure of at least 100 GPa or more to be achieved, which is generally difficult to achieve under very harsh conditions in the laboratory. This gave rise to the original intention of researchers to explore high‐temperature superconductors at ambient or near ambient pressure. To achieve this goal, more novel systems and structures are being studied.

Due to the pre‐compression effect of the crystal structure, hard or superhard materials are considered as potential high‐temperature superconductors at ambient pressure. The superconductivity of numerous hard and superhard materials at ambient pressure has been experimentally observed or theoretically predicted. The superconductivity with *T*_*c*_ ∼ 38 K was observed in Cs‐doped C_60_.^[^ ^7^ ^]^ The *T*_*c*_ of diamond was predicted to reach 55 K when the doping concentration of boron is in the range of 20–30%.^[^ ^8^ ^]^ In clathrate B‐N compounds at ambient pressure, the *T*_*c*_ of La(BN)_5_ was predicted as 69 K,^[^ ^9^ ^]^ and the *T*_*c*_ of Al(BN)_3_ with sodalite structure was predicted to be 72 K.^[^ ^10^ ^]^ Furthermore, higher *T*_*c*_ at ambient pressure was predicted in clathrate B–C compounds, such as 83 K in Rb_0.4_Sr_0.6_(BC)_3_^[^ ^11^ ^]^ and 88 K in K_0.5_Pb_0.5_(BC)_3_.^[^ ^12^ ^]^ However, among numerous hard and superhard materials, there are few reports of *T*_*c*_ above 100 K at ambient pressure. In our previous study,^[^ ^13^ ^]^ by doping diamond with H element, we achieved the superconductivity of 117 K at 5 GPa, which has already approached the ambient‐pressure high‐temperature superconductivity. Cubic boron nitride (*c*‐BN) is another well‐known superhard material with diamond‐like structure. Solozhenko et al.,^[^ ^14^, ^15^ ^]^ and Maki et al.,^[^ ^16^ ^]^ claimed that, if diamond is a metastable phase, *c*‐BN could be the stable form of BN at ambient pressure conditions. Thus, the exploration of possible ambient‐pressure high‐temperature superconductivity in *c*‐BN is very meaningful. In this work, therefore, we report the structure, electronic states, dynamics, and electron–phonon interactions of doped *c*‐BN at ambient pressure based on the first‐principles calculations.

## 2 Results and Discussion

At ambient pressure, *c*‐BN has the $F\bar{4}3m$ group‐space (Figure S1a, Supporting Information), and our optimized lattice constant is *a* = 3.623 Å, which is consistent with the previous study.^[^ ^17^ ^]^ Pure *c*‐BN is an indirect band gap semiconductor with a band gap of 4.45 eV (Figure S2, Supporting Information), although theoretical predictions are slightly different from the experimental observations.^[^ ^18^, ^19^ ^]^ Within the framework of BCS theory, to achieve superconductivity, the system should first be transformed into a metal. Doping is an effective means to achieve this transformation, which can preserve the structural characteristics of the *c*‐BN material as much as possible. Thus, we doped other atoms into the interstitial sites of the crystal lattice. There are two kinds of interstitial sites in *c*‐BN, octahedral and tetrahedral interstices (Figure S1b,c, Supporting Information). Similar to the doped diamond,^[^ ^13^ ^]^ we found that the doped *c*‐BN is more stable when the dopant atom is on the interstitial site of an octahedron formed by B and N atoms. After trying the doping of 27 elements (Al, As, Ba, Be, Bi, Br, Ca, Cl, F, H, Li, K, Mg, Na, O, P, Pb, Po, S, Sb, Sc, Se, Si, Sr, Te, Tl, and Y), we found that only H and Li with smaller radii can be doped into *c*‐BN. The energy difference between dopant atom in octahedral and tetrahedral interstices is ≈0.205 eV per atom for HB_4_N_4_ or 0.012 eV per atom for LiB_4_N_4_, respectively. Hence, we only considered the stoichiometries of HB_4_N_4_ and LiB_4_N_4_.

**Figure 1:**
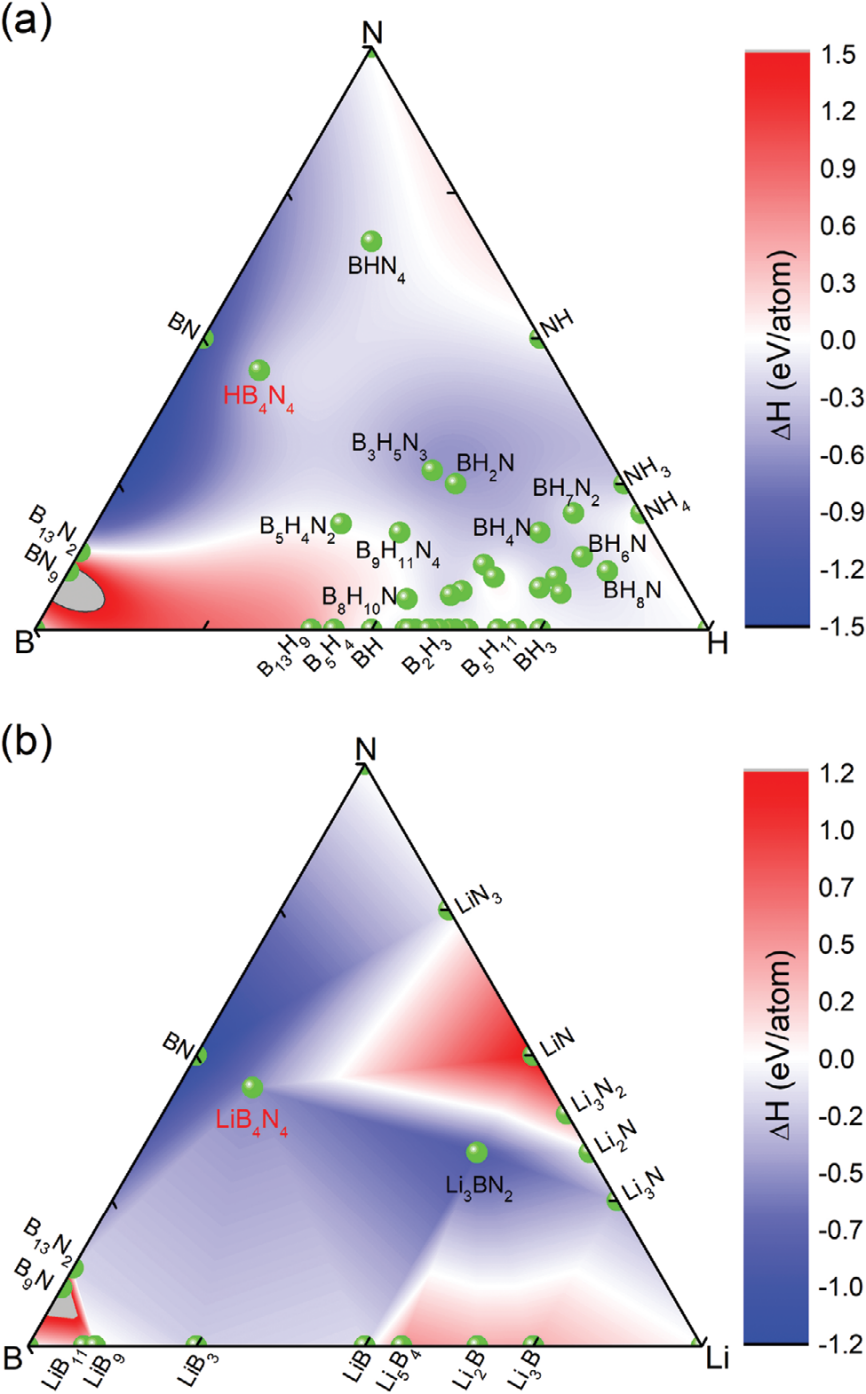
Ternary phase diagrams of H‐B‐N a) and Li‐B‐N b) systems at ambient pressure. Color bar implies the magnitude of enthalpy of formation.

Inserting one H (or Li) atom into octahedral interstice, the space group of system transforms into $P\bar{4}3m$ from $F\bar{4}3m$. Doping leads to the slight expansion of crystal volume. The lattice constant is *a* = 3.705 Å for one H atom doped *c*‐BN (HB_4_N_4_) and *a* = 3.791 Å for one Li atom doped *c*‐BN (LiB_4_N_4_), respectively. The binding energies are 0.730 eV for HB_4_N_4_ and 0.777 eV for LiB_4_N_4_, respectively. Additionally, the calculated elastic moduli of *C*_11_, *C*_12_, and *C*_44_ are 611, 250, and 136 GPa for HB_4_N_4_, and 759, 144, and 202 GPa for LiB_4_N_4_, respectively. These elastic moduli satisfy the criteria of elastic stability of *C*_11_ − *C*_12_ > 0, *C*_11_ + 2*C*_12_ > 0, and *C*_44_ > 0. Thus, doped *c*‐BN is mechanically stable. To examine the phase stability, we have calculated the enthalpies of the formation of numerous binary and ternary phases. **Figure** **1** shows the ternary phase diagrams of H–B–N and Li–B–N systems at ambient pressure. This diagram was constructed considering reported binary and ternary phase structures.^[^ ^20^ ^]^ On the one hand, the ternary phase diagram can reflect the stability of the target compounds (HB_4_N_4_ and LiB_4_N_4_) relative to the elemental phases (H, Li, B, and N), binary phases (H–B, H–N, Li–B, Li–N, and B–N), and other ternary phases. On the other hand, it also suggests possible experimental synthesis schemes. It was found that both HB_4_N_4_ and LiB_4_N_4_ are metastable compared with pure *c*‐BN phase. However, the stability of HB_4_N_4_ and LiB_4_N_4_ is better than most other ternary and binary phases. In addition, fixing the stoichiometries of HB_4_N_4_ and LiB_4_N_4_, the thermodynamic stability has been further investigated by comparing $P\bar{4}3m$ phase with other possible phases (Figures S3 and S4, Supporting Information). HB_4_N_4_ and LiB_4_N_4_ with $P\bar{4}3m$ space‐group are both thermodynamically metastable from the enthalpies of formation, although $P\bar{4}3m$ phase is not the most stable one. The metastable phase does not necessarily mean that it does not exist, as the energy barrier between the metastable phase and the globally stable phase can usually suppress the occurrence of structural phase transition. From the perspective of experimental synthesis, Figure 1 suggests that the combination of some elemental phases and binary or ternary phases under certain conditions may form the target compound of HB_4_N_4_ and LiB_4_N_4_.

**Figure 2:**
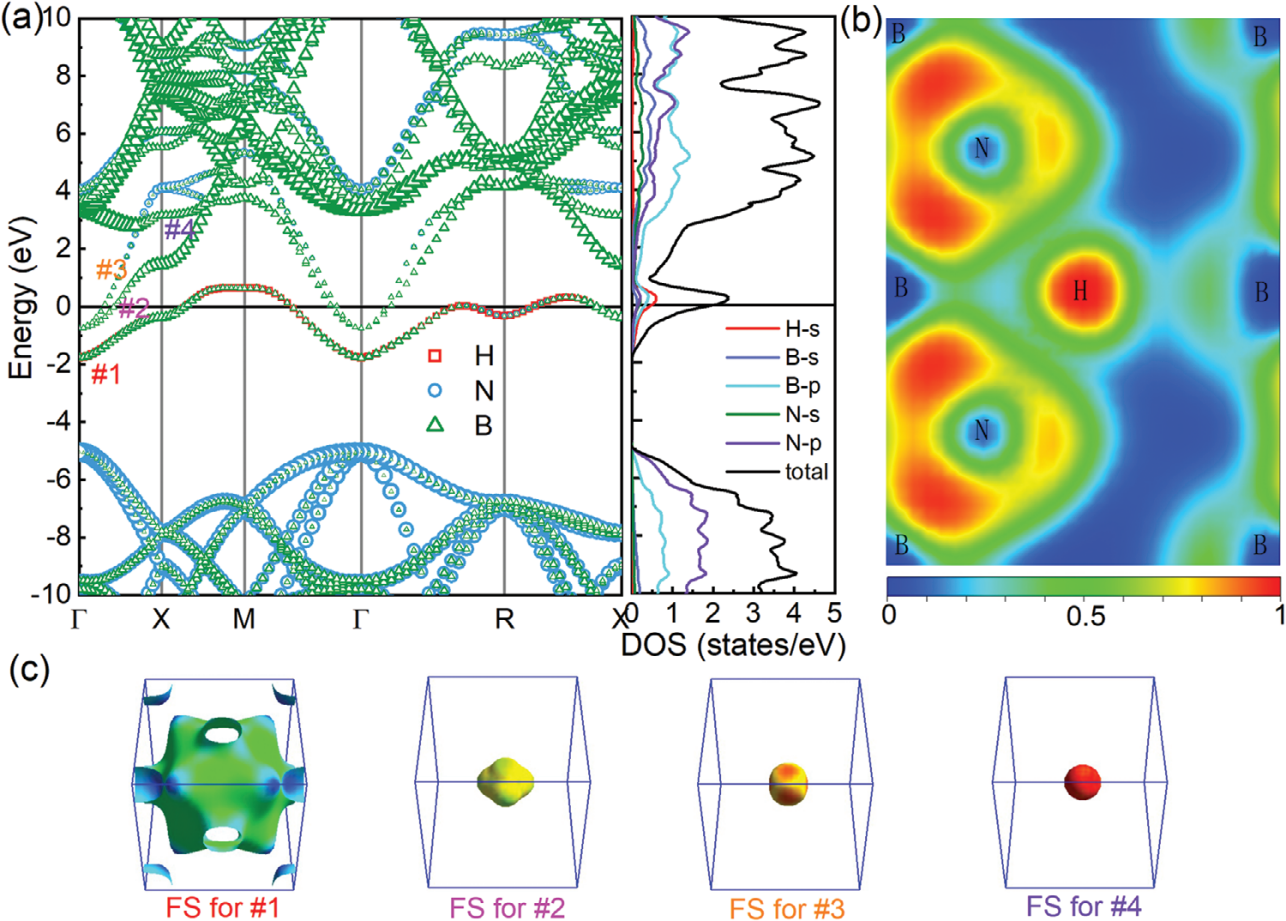
a) Characterized band structures and total and projected DOS of $P\bar{4}3m$‐HB_4_N_4_ at ambient pressure. Zero energy denotes the Fermi level. In band structures, the symbol size implies the weight of different electronic states in the hybrid band. Four bands crossing Fermi level are marked as #1, #2, #3, and #4. b) 2D ELF on (101) plane. c) Fermi surfaces corresponding to band #1, #2, #3, and #4.

The doping of H and Li can make *c*‐BN exhibit the metallic feature, which is verified by two functional methods (Figure S5, Supporting Information). **Figure** **2** shows the band structures along high‐symmetry *k*‐point paths, total and projected density of states (DOS), 2D electron localization function (ELF) on (101) plane, and Fermi surface sheets of $P\bar{4}3m$‐HB_4_N_4_ at ambient pressure. The introduction of H atom causes Fermi level shifting toward higher level and passing through the conduction bands. As shown in Figure 2, both conduction band minimum (CBM) and valence band maximum (VBM) are formed by the hybridization between B and N, but CBM is mainly contributed by B‐*2p* electronic states, while VBM is mainly contributed by N‐*2p* states. It is worth noting that a new hybridized band appears in the band gap of *c*‐BN. It can be seen from ELF that there is a strong hybridization between H and N. Combining band structures and projected DOS, we found that H dominates the newly added hybridization band. Hence, the metallization of $P\bar{4}3m$‐HB_4_N_4_ mainly originates from the hybridization between H and nearby N atoms. Further analyzing the causes of metallization, we also observed the electronic structures of three thermodynamically relatively stable phases of *Pnc*2‐HB_4_N_4_, *P*6_3_ *mmc*‐HB_4_N_4_, and *P*4*cc*‐HB_4_N_4_ which do not possess the *c*‐BN crystal structure characteristics (Figure S3, Supporting Information). The band structures and the projected DOS (Figure S6, Supporting Information) clearly indicate that even in systems without *c*‐BN structural features, the metallization also comes from hybridization of H and N, and new hybridization bands appear near the Fermi level. As shown in Figure 2, in $P\bar{4}3m$‐HB_4_N_4_, four bands marked as band #1, #2, #3, and #4 cross over Fermi level, forming electron or hole‐like Fermi surfaces (FS). Band #1 mainly forms the hole‐like FS sheets, while other three bands form the electron‐like FS sheets around Γ point. At Fermi level, the DOS value is ≈1.8 states per eV, which is significantly higher than 0.64 states per eV of H‐doped diamond at 5 GPa,^[^ ^13^ ^]^ and larger than those of hydrides, such as 0.45 states per eV of H_3_S at 200 GPa^[^ ^21^ ^]^ and 0.74 states per eV of LaH_10_ at 250 GPa.^[^ ^22^ ^]^ Three stable phases also generated high DOS value at Fermi level, which are 1.13, 0.90, and 2.01 states per eV for *Pnc*2‐HB_4_N_4_, *P*6_3_ *mmc*‐HB_4_N_4_, and *P*4*cc*‐HB_4_N_4_, respectively.

**Figure 3:**
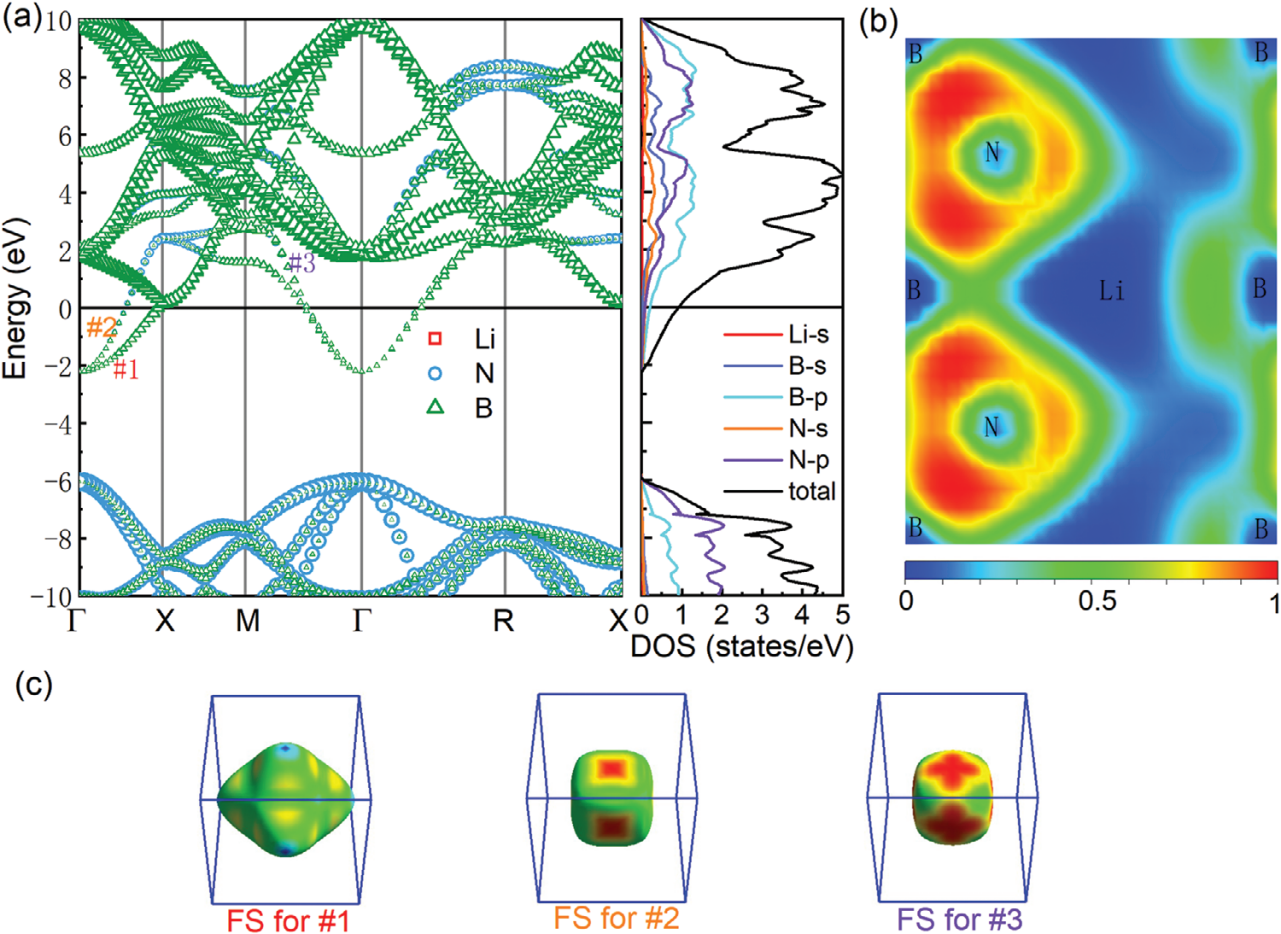
a) Characterized band structures and total and projected DOS of $P\bar{4}3m$‐LiB_4_N_4_ at ambient pressure. Zero energy denotes the Fermi level. In band structures, the symbol size implies the weight of different electronic states in the hybrid band. Three bands crossing Fermi level are marked as #1, #2, and #3. b) 2D ELF on (101) plane. c) Fermi surfaces corresponding to band #1, #2, and #3.

Compared with H doping, $P\bar{4}3m$‐LiB_4_N_4_ exhibits a different band structure. In **Figure** **3**, although Li doping also results in Fermi level shifting toward higher levels, it does not introduce new energy bands near Fermi level. It seems that the energy band has only shifted relative to Fermi level. Similarly, in the relatively stable Li– B– N phases of *P*6_3_ *mmc*‐LiB_4_N_4_, *I*422‐LiB_4_N_4_, and *Pm*3‐LiB_4_N_4_, the electronic states of Li are lacking near the Fermi level, and the electronic structures (Figure S7, Supporting Information) also exhibits obvious charge transfer characteristics, suggesting that metallization mainly comes from the charge transfer. In $P\bar{4}3m$‐LiB_4_N_4_, three bands cross over the Fermi level and form the electron‐like FS sheets around Γ point. The electronic states at Fermi level are mainly contributed by B‐2*p* electrons. The DOS value at Fermi level is 0.94 states per eV, which is obviously less than 1.8 states per eV of HB_4_N_4_. From ELF in Figure 3, there is visible ionic bonding characteristics between Li and N (or B). The transferred charge is ≈0.68 e/Li from Li to N. Thus, we point out that the metallization of LiB_4_N_4_ mainly comes from the shift of Fermi level induced by charge transfer.

**Figure 4:**
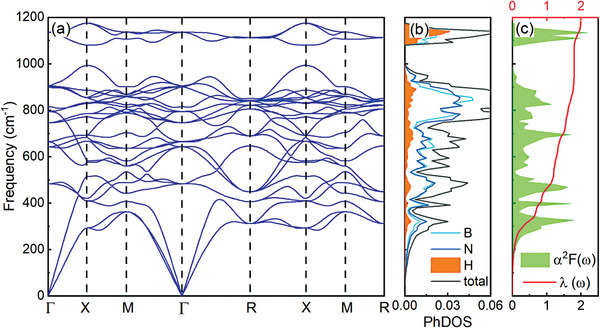
a) Phonon spectra, b) total and projected PhDOS, c) Eliashberg function α^2^ *F*(ω), and EPC integral λ(ω) of $P\bar{4}3m$‐HB_4_N_4_ at ambient pressure.

In addition to electronic structures, phonon characteristics are another important property for exploring the possible superconductivity of doped *c*‐BN. We have calculated the phonon spectra of HB_4_N_4_ with *Pnc*2, *P*6_3_ *mmc*, *P*4*cc*, and $P\bar{4}3m$ space group as well as LiB_4_N_4_ with *P*6_3_ *mmc*, *I*422, *Pm*3, and $P\bar{4}3m$ at ambient pressure. The existence of imaginary frequencies (Figure S8, Supporting Information) implies that the thermodynamically relatively stable *Pnc*2‐, *P*6_3_ *mmc*‐, *P*4*cc*‐HB_4_N_4_ and *P*6_3_ *mmc*‐, *I*422‐, *Pm*3‐LiB_4_N_4_ are dynamically unstable at ambient pressure. **Figure** **4** shows phonon spectra and phonon density of states (PhDOS) of $P\bar{4}3m$‐HB_4_N_4_ at ambient pressure. The absence of imaginary frequencies implies that $P\bar{4}3m$‐HB_4_N_4_ is dynamically stable at ambient pressure. In $P\bar{4}3m$‐HB_4_N_4_, the phonon vibration frequency covers the range of 0 − 1112 cm^−1^, which is similar to the situation of carbides^[^ ^13^ ^]^ and BN compounds,^[^ ^9^, ^10^, ^11^, ^12^ ^]^ but less than the phonon frequency range of hydrogen‐rich systems.^[^ ^21^, ^22^ ^]^ Combining the projected PhDOS on elements with phonon spectra, we found that the vibration frequencies of the three atoms are also distributed throughout the entire frequency range.

**Figure 5:**
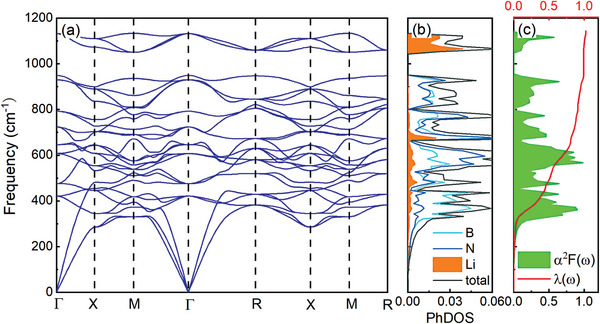
a) Phonon spectra, b) total and projected PhDOS, c) Eliashberg function α^2^ *F*(ω), and EPC integral λ(ω) of $P\bar{4}3m$‐LiB_4_N_4_ at ambient pressure.

The Eliashberg spectral function (α^2^ *F*(ω))^[^ ^23^ ^]^ of $P\bar{4}3m$‐HB_4_N_4_ was calculated and shown in Figure 4c. With the help of α^2^ *F*(ω), the electron–phonon coupling (EPC) constant λ and the logarithmic average of phonon frequency (ω_log_) are obtained. The total EPC constant λ is 1.98 for $P\bar{4}3m$‐HB_4_N_4_ at ambient pressure, which is a strong electron–phonon interaction. From the EPC integral λ(ω) shown in Figure 4c, almost all phonon states contribute to the EPC integration, but in the frequency range of 275 − 500 cm^−1^, the trend of EPC integration increasing is obvious, indicating that there are more soft phonon modes in this range. The calculated ω_log_ of $P\bar{4}3m$‐HB_4_N_4_ is 688.0 K. Furthermore, *T*_*c*_ was estimated by using the Allen‐Dynes‐corrected McMillan equation,^[^ ^24^ ^]^ based on the Coulomb pseudopotential (µ^⋆^) in the range of 0.1 − 0.13. The *T*_*c*_ of $P\bar{4}3m$‐HB_4_N_4_ is predicted as 122.6 K for µ^⋆^ = 0.1 or 111.5 K for µ^⋆^ = 0.13. Thus, we achieved ambient‐pressure superconductivity exceeding 100 K in H‐doped *c*‐BN. $P\bar{4}3m$‐LiB_4_N_4_ exhibits slightly different phonon spectra shown in **Figure** **5**. The soft phonon modes reduces in $P\bar{4}3m$‐LiB_4_N_4_, especially around *R* point. Although a slightly high ω_log_ of 716.9 K is obtained, the intensity of α^2^ *F*(ω) function of $P\bar{4}3m$‐LiB_4_N_4_ is significantly weaker than that of $P\bar{4}3m$‐HB_4_N_4_. As a result, the λ and *T*_*c*_ are 1.05 and 58.5 K for µ^⋆^ = 0.1 (50.7 K for µ^⋆^ = 0.13) at ambient pressure, respectively. H‐doped and Li doped *c*‐BN exhibit different superconducting transition temperatures, and the differences in superconductivity can be understood from the electronic structures and phonon properties. As mentioned above, Li insertion into *c*‐BN exhibits ionic bonding characteristics, while H insertion into *c*‐BN involves more covalent bonding, resulting in different electronic density of states near the Fermi level. H doping leads to a higher electronic density of states at Fermi level. From the perspective of the interaction between phonon and electron, the covalent bonding in H‐doped *c*‐BN generates stronger Eliashberg functions, resulting in stronger EPC constant. This also suggests that it is crucial to obtain a stronger Eliashberg function in driving stronger superconductivity in doped *c*‐BN.

**Figure 6:**
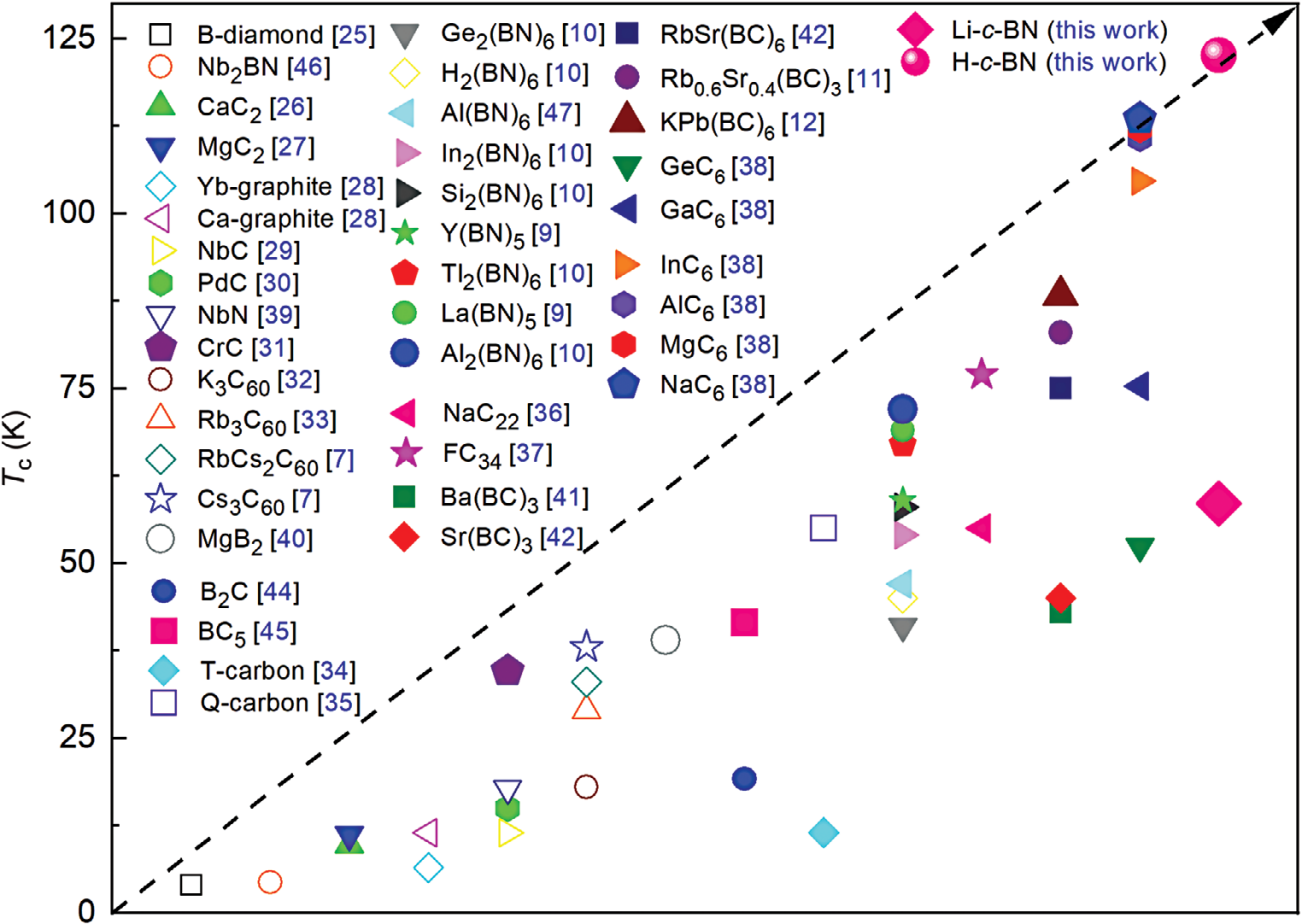
Comparison of *T*_*c*_'s of some carbides, nitrides, borides, carbon–boron compounds, and boron‐nitrogen compounds with ambient‐pressure superconductivity. Hollow symbols represent the measured experimental results and solid symbols represent the results from theoretical calculations.

Although the *T*_*c*_ of doped *c*‐BN is lower than those of some hydrides, such as H_3_S^[^ ^21^ ^]^ and LaH_10_,^[^ ^22^ ^]^ it does achieve superconductivity exceeding 100 K at ambient pressure. Especially, *T*_*c*_ of H‐doped *c*‐BN is generally higher than that of some carbides,^[^ ^7^, ^25^, ^26^, ^27^, ^28^, ^29^, ^30^, ^31^, ^32^, ^33^, ^34^, ^35^, ^36^, ^37^, ^38^ ^]^ nitrides,^[^ ^39^ ^]^ borides,^[^ ^40^ ^]^ carbon‐boron compounds,^[^ ^11^, ^12^, ^41^, ^42^, ^43^, ^45^ ^]^ and boron–nitrogen compounds.^[^ ^9^, ^10^, ^46^, ^47^ ^]^ As shown in **Figure** **6**, the systems mentioned here are mostly hard or superhard materials. From previous results, it was found that the system has higher *T*_*c*_ when C, B, and N atoms form cage like structures. For example, the *T*_*c*_ increases from 38 K of Cs_3_C_60_^[^ ^7^ ^]^ to 55 K of Q‐carbon^[^ ^35^ ^]^ and NaC_22_,^[^ ^36^ ^]^ 72 of Al_2_(BN)_6_,^[^ ^10^ ^]^ 77 K of FC_34_, then to 88 K of KPb(BC)_6_,^[^ ^12^ ^]^ and then to ≈100 K of *M*C_6_ (*M* = Na, Mg, Al, In, etc.)^[^ ^38^ ^]^. In this study, we have predicted the ambient‐pressure superconductivity exceeding 100 K in non‐cage‐structural light‐weight‐element system.

Finally, we go back to the discussion of stability and possible experimental preparation. Although the doped *c*‐BN with $P\bar{4}3m$ symmetry is not thermodynamically stable, its metastable properties suggest the existence of some possible synthesis pathways. As shown in Figure 1, from the ternary phase diagram, it can be seen that the target compound can be synthesized from the elemental phase. At the same time, since the stability of the target compound is higher than that of most binary and some ternary phases, it can also be synthesized using the binary and ternary phases. For example, when synthesizing the target compound by combining H_2_ with *c*‐BN, we performed a calculation of the enthalpy of formation as a function of pressure (Figure S9, Supporting Information). The results showed that the formation energy of $P\bar{4}3m$‐HB_4_N_4_ above 165 GPa was lower than the sum of the formation energies of H_2_ and *c*‐BN. Specifically, compared with the other three thermodynamically more stable phases at ambient pressure, it was found that the stability of $P\bar{4}3m$‐HB_4_N_4_ gradually improved at high pressure, and the results showed that $P\bar{4}3m$‐HB_4_N_4_ is the most stable phase above ≈185 GPa. It means that $P\bar{4}3m$‐HB_4_N_4_ can be synthesized at high pressures above 185 GPa and then released at low pressures. The change from H_2_ molecule to H atom can be achieved through laser heating. In fact, in high‐pressure hydrogen compound synthesis experiments, by‐products of *c*‐BN and H_2_ are often produced in diamond anvil cells, which is the basis for further high‐pressure synthesis of H‐doped *c*‐BN.^[^ ^48^ ^]^ Similarly, $P\bar{4}3m$‐LiB_4_N_4_ compound can also be synthesized by combining Li metal with *c*‐BN (Figure S10, Supporting Information). And above 148 GPa, $P\bar{4}3m$‐LiB_4_N_4_ exhibits the best stability.

In summary, exploring the ambient‐pressure high‐temperature superconductivity, we have studied crystal structures and electronic states of superhard material *c*‐BN doped by H and Li atom based on the first‐principles calculations and estimated *T*_*c*_ by calculating EPC and using corrected McMillan equation. The insertions of H and Li lead to the metallization transition of *c*‐BN. The hybridization between H and N drives the metallization of H‐doped *c*‐BN, while the charge transfer from Li to N causes the metallization of Li doped *c*‐BN. For thus superhard material, doped *c*‐BN exhibits the good superconductivity at ambient pressure. *T*_*c*_ of $P\bar{4}3m$‐LiB_4_N_4_ is 58 K. Remarkably, $P\bar{4}3m$‐HB_4_N_4_ achieves the high‐temperature superconductivity with *T*_*c*_ ∼ 122 K. Compared to charge transfer, the hybridization between H and BN results in stronger EPC interactions. The results show that it is feasible to explore the ambient‐pressure higher‐*T*_*c*_ superconductivity in hard or superhard materials.

## 3 Experimental Section

The structural optimizations and self‐consistent energy calculation on pristine and doped *c*‐BN were carried out by employing QUANTUM ESPRESSO package (QE).^[^ ^49^, ^50^ ^]^ The exchange‐correlation functional of generalized gradient approximation (GGA) in the version of Perdew–Burke–Ernzerhof (PBE)^[^ ^51^ ^]^ and projector‐augmented wave (PAW) pseudopotentials^[^ ^52^ ^]^ were adopted. The plane wave cutoff energy was set as 80 Ry. During the optimization process, convergence thresholds were set as 10^−5^ eV in energy and 10^−3^ eV Å^−1^ in force. The *k*‐point interval distribution of Monkhorst–Pack was 0.01 Å^−1^ for structural optimization and self‐consistent energy calculation.

The phonon frequencies and electron‐phonon interactions of pristine and doped *c*‐BN were calculated with the help of QE package. The PAW‐type pseudopotentials for H (H.pbe‐kjpaw‐psl.1.0.0.UPF), B (B.pbe‐n‐kjpawpsl.1.0.0.UPF), and N (N.pbe‐n‐kjpaw‐psl.1.0.0.UPF) were used in QE code. The normal *k*‐point grid of Monkhorst‐Pack of 16 × 16 × 16 and an irreducible *q*‐point grid of 8 × 8 × 8 were used in the calculation of the electron–phonon interaction matrix element.

Based on the calculated frequency, the electronic DOS at Fermi level, and the phonon linewidth γ_*Q*ν_, the Eliashberg spectral function α^2^ *F*(ω) of H‐doped c‐BN was calculated by^[^ ^53^ ^]^

(1) $$\alpha^{2}F\left(\omega \right)=\frac{1}{2\pi N(E_{F})}\sum_{Q\nu }\frac{\gamma_{Q\nu }}{\omega_{Q\nu }}\delta \left(\omega -\omega_{Q\nu }\right)$$

With help of α^2^ *F*(ω), the EPC constant λ and the logarithmic average of phonon frequency ω_log_ are obtained by

(2) $$\lambda =2\int_{0}^{\infty }\frac{\alpha^{2}F\left(\omega \right)}{\omega }d\omega$$

and

(3) $$\omega_{\log}=\exp\left[\frac{2}{\lambda }\int_{0}^{\infty }\frac{\alpha^{2}F\left(\omega \right)\log\left(\omega \right)}{\omega }d\omega )\right]$$

Furthermore, based on the calculated λ and ω_log_, *T*_*c*_ was estimated by the Allen–Dynes–corrected McMillan equation,^[^ ^24^ ^]^ expressed as:

(4) $$T_{c}=f_{1}f_{2}\frac{\omega_{\log}}{1.2}\exp\left[-\frac{1.04(1+\lambda )}{\lambda -\mu^{*}\left(1+0.62\lambda \right)}\right]$$

where µ^⋆^ represents the Coulomb pseudopotential which is generally in the range of 0.1 − 0.13 for light‐weight element compounds. The factor *f*_1_ *f*_2_ depends on the λ, µ^⋆^, ω_log_, and mean square frequency ($\bar{\omega_{2}}$).^[^ ^24^ ^]^

## Conflict of Interest

The authors declare no competing interests.

## Author Contributions

H.‐B.D., and R.N., contributed equally to this work. X.J.C., H.Q.L., and G.H.Z. conceived this project. H.B.D., R.N., S.A.L., and Y.M.L. carried out the theoretical calculations. R.N., H.B.D., X.J.C., H.Q.L., and G.H.Z. analyzed the data. R.N., X.J.C., H.Q.L., and G.H.Z. wrote and revised the paper with the inputs from other authors. All authors discussed the results. The manuscript reflects the contributions of all authors.

## Supporting information

Supporting Information

## Data Availability

The data that support the findings of this study are available in the supplementary material of this article.
