# Supplementary material for: Hydrogen‐Doped c‐BN as a Promising Path to High‐Temperature Superconductivity Above 120 K at Ambient Pressure
Source: Adv Sci (Weinh). 2024 Oct 7;11(44):2408275. doi: 10.1002/advs.202408275 (PMC11600296; doi:10.1002/advs.202408275)
Supplement: Supplementary file 1 — Supporting Information [file ADVS-11-2408275-s001.docx]

Supporting Information

**Hydrogen-doped *c*-BN as a promising path to high-temperature superconductivity above 120 K at ambient pressure**

Han-Bin Ding,^1,2,7^ Rui Niu,^1,2,7^ Shen-Ao Li,^1,3^ Ying-Ming Liu,^1,3^ Xiao-Jia Chen,^4,5,*^ Hai-Qing Lin,^6,*^ and Guo-Hua Zhong,^1,3,*^

*^1^Shenzhen Institute of Advanced Technology, Chinese Academy of Sciences, Shenzhen 518055, China*

*^2^Nano Science and Technology Institute, University of Science and Technology of China, Suzhou 215123, China*

*^3^University of Chinese Academy of Sciences, Beijing 100049, China*

*^4^Department of Physics and Texas Center for Superconductivity, University of Houston, Houston, TX 77204, USA*

*^5^School of Science, Harbin Institute of Technology, Shenzhen 518055, China*

*^6^School of Physics, Zhejiang University, Hangzhou 310058, China*

*^7^These authors contributed equally: Han-Bin Ding, Rui Niu*

*^*^Corresponding authors: xjchen@uh.edu (Xiao-Jia Chen), hqlin@zju.edu.cn (Hai-Qing Lin), gh.zhong@siat.ac.cn (Guo-Hua Zhong)*

**1. Crystal structures of pure and doped *c*-BN at ambient pressure**

**
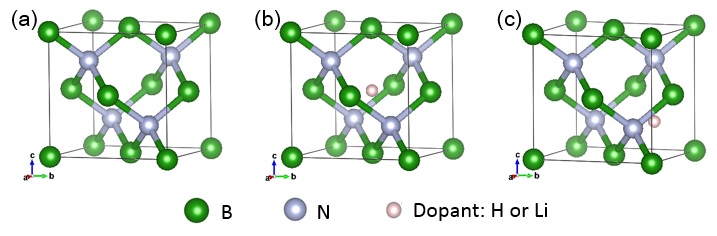
**

**Fig. S**1 (a) Crystal structure of pure *c*-BN. (b) Crystal structure of doped *c*-BN with dopant atom being at the octahedral interstice. (c) Crystal structure of doped *c*-BN with dopant atom being at a tetrahedral interstice.

**2. Band structures of pure *c*-BN at ambient pressure**

**
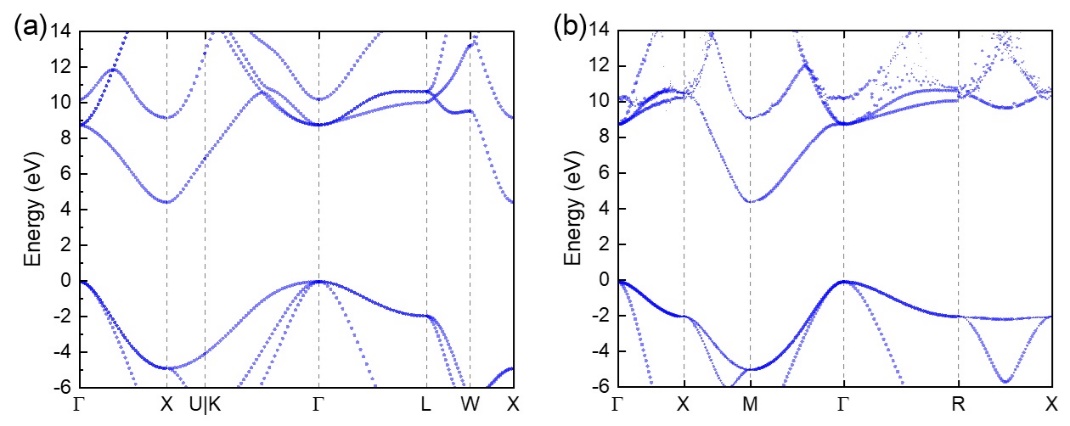
**

**Fig. S2** Calculated band structures along high symmetry *k*-point paths of pure *c*-BN at ambient pressure. (a) Pure *c*-BN was set as $F\bar{4}3m$ space-group. (b) Pure *c*-BN was set as $P\bar{4}3m$ space-group. High symmetry *k*-points in (a): Γ(0, 0, 0), X(0.5, 0, 0.5), U(0.625, 0.25, 0.625), K(0.375, 0.375, 0.75), L(0.5, 0.5, 0.5), W(0.5, 0.25, 0.75); and high symmetry k-points in (b): Γ(0, 0, 0), X(0, 0.5, 0), M(0.5, 0.5, 0), R(0.5, 0.5, 0.5), X(0, 0.5, 0).

**3. Possible phase structures of HB_4_N_4_ and LiB_4_N_4_ at ambient pressure**

Fixing the chemical ratio of HB_4_N_4_ and LiB_4_N_4_, we searched for possible crystal structures at ambient pressure by employing the CALYPSO [1,2] code based on the particle swarm optimization method. The relatively stable 8 structures and their lattice parameters are shown in the following figure, including $P\bar{4}3m$-HB_4_N_4_ and $P\bar{4}3m$-LiB_4_N_4_. From the calculated enthalpies of formation (*E*_f_) these phases, $P\bar{4}3m$-HB_4_N_4_ and $P\bar{4}3m$-LiB_4_N_4_ are both thermodynamically metastable at ambient pressure.

| **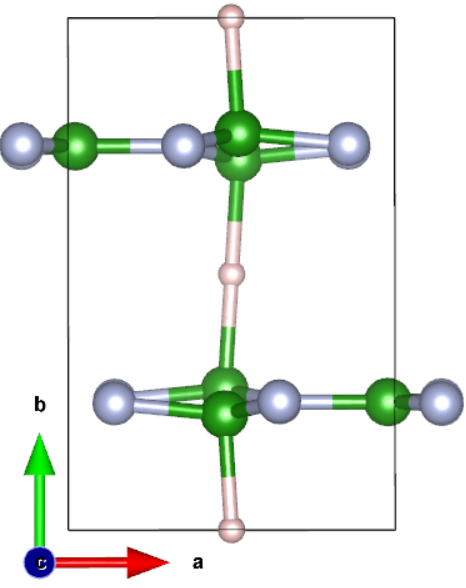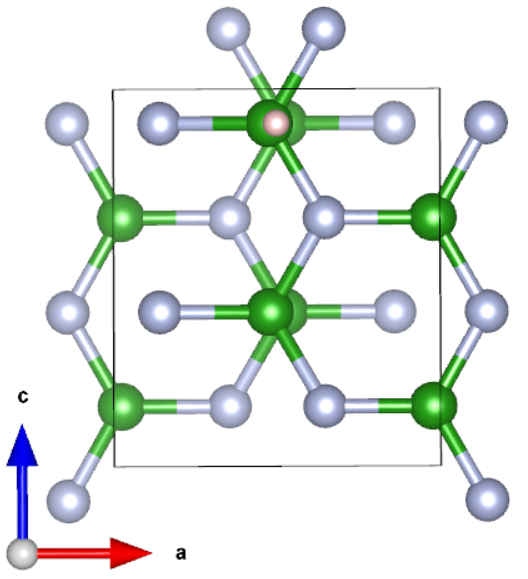**  *Pnc*2-HB_4_N_4_  ***E*_f_ = -7.951 eV/atom**  a=4.374 Å, b=6.853 Å, c=5.050 Å | 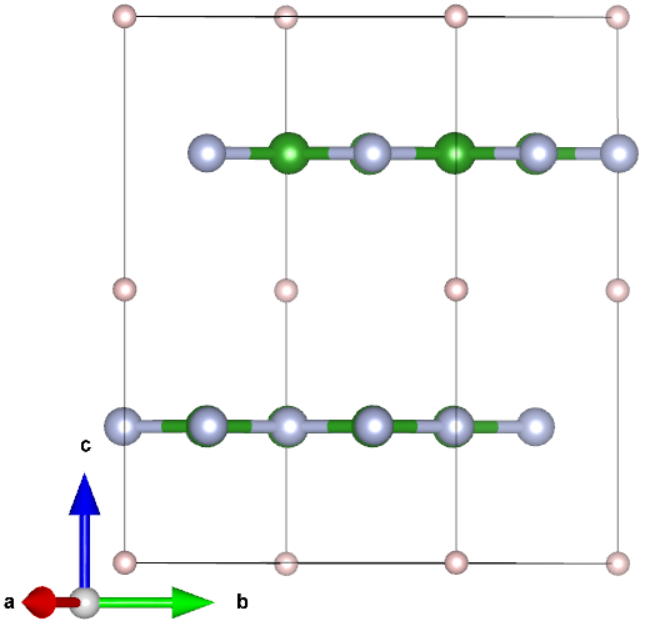 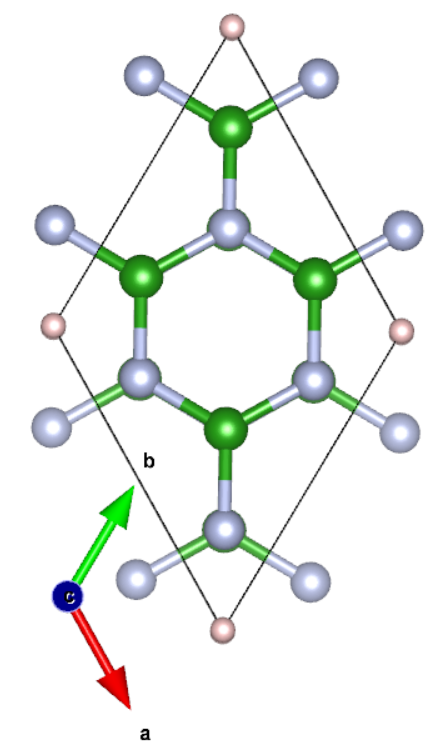  *P*6_3_/*mmc*-HB_4_N_4_  ***E*_f_ = -7.882 eV/atom**  a=5.025 Å, b=5.025 Å, c=8.276 Å |
| --- | --- |
| **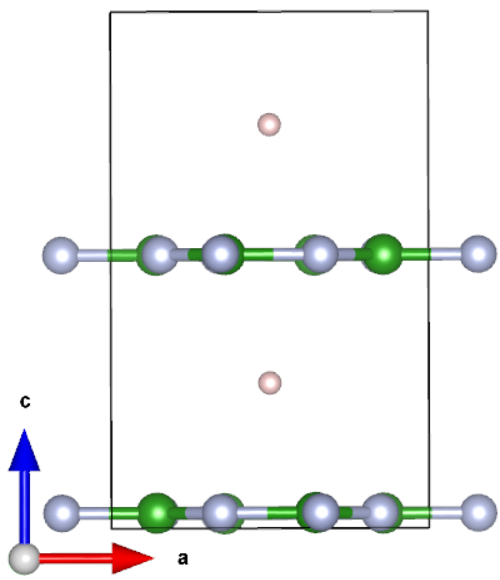 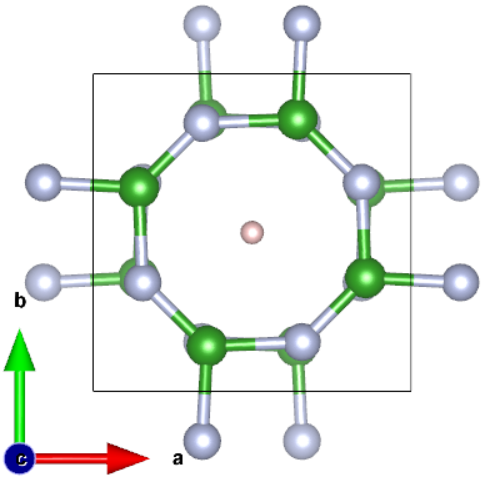**  *P*4*cc*-HB_4_N_4_  ***E*_f_ = -7.584 eV/atom**  a=4.940 Å, b=4.940 Å, c=8.030 Å | **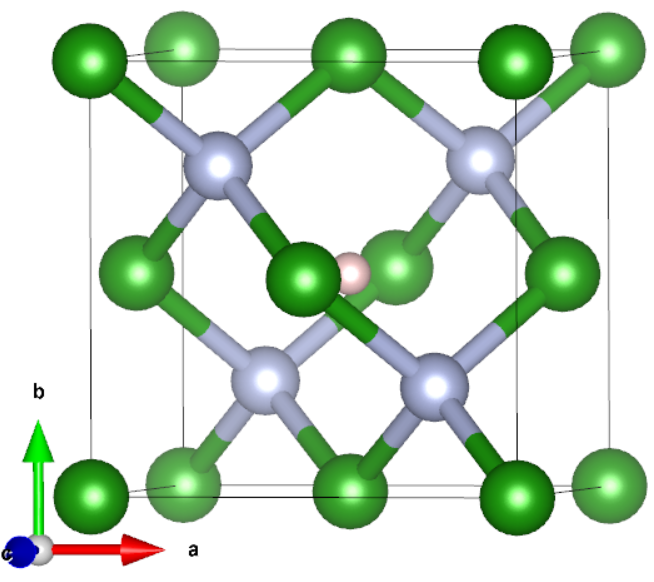**  $P\bar{4}3m$-HB_4_N_4_  ***E*_f_ = -7.400 eV/atom**  a=3.705 Å, b=3.705 Å, c=3.705 Å |
| **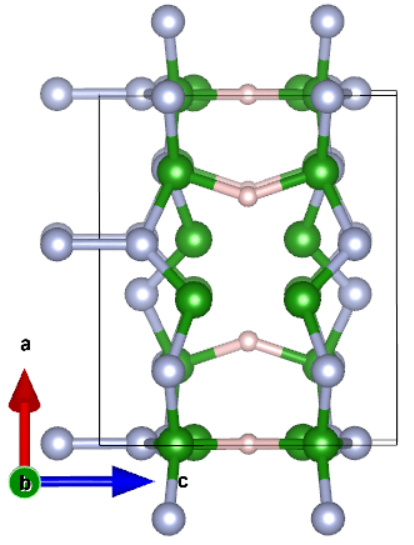 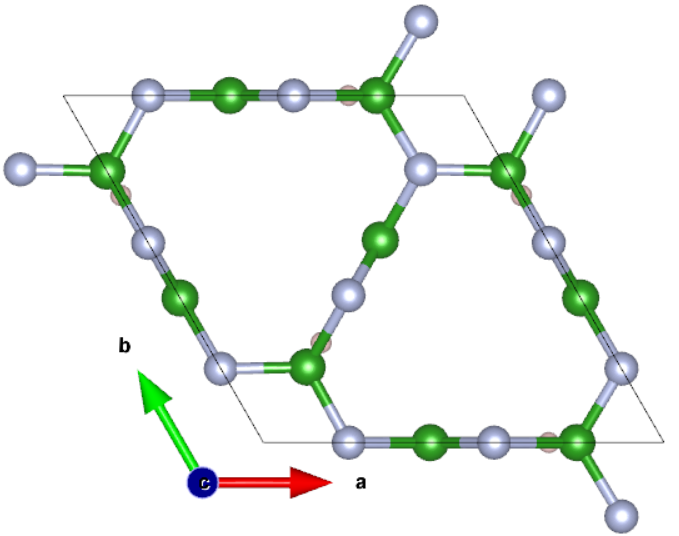**  $P\bar{6}2m$-HB_4_N_4_  ***E*_f_ = -7.340 eV/atom**  a=6.895 Å, b=6.895 Å, c=5.118 Å | **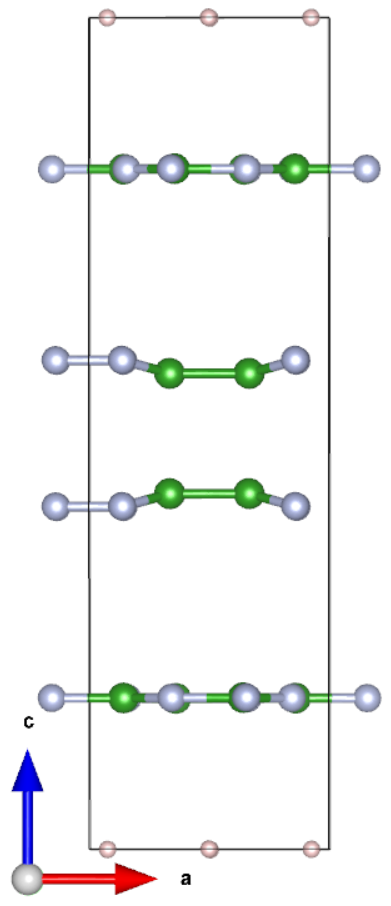 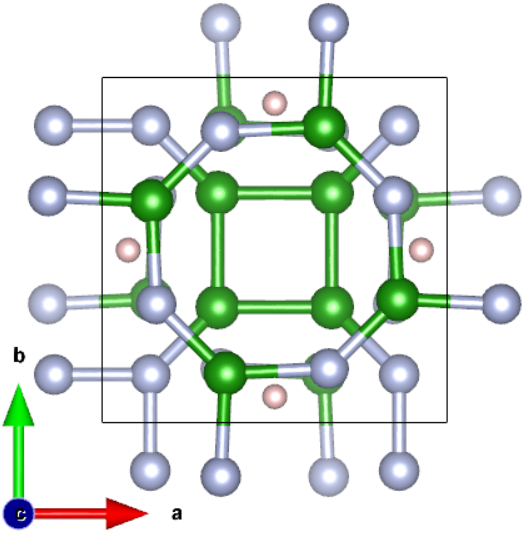**  *P*422-HB_4_N_4_  ***E*_f_ = -7.253 eV/atom**  a=4.998 Å, b=4.998 Å, c=17.324 Å |
| **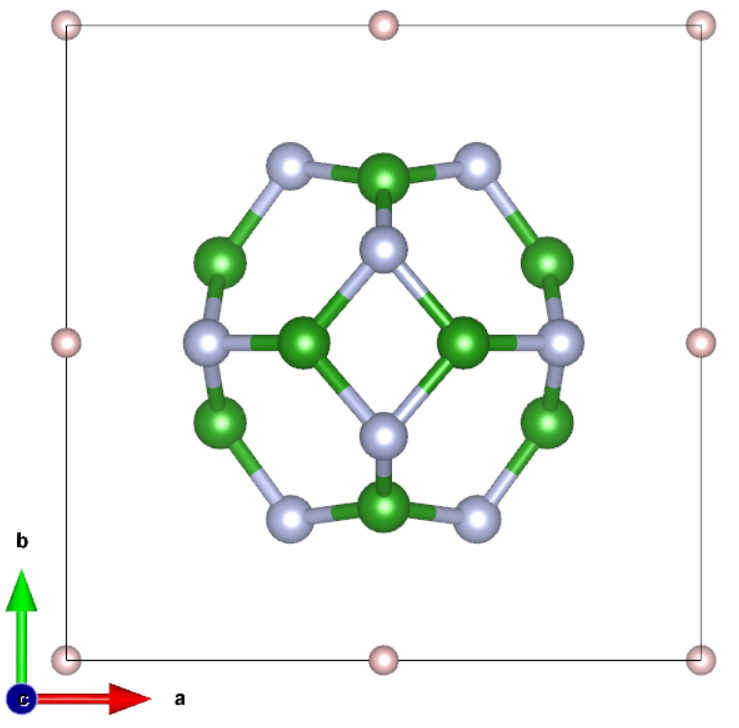** **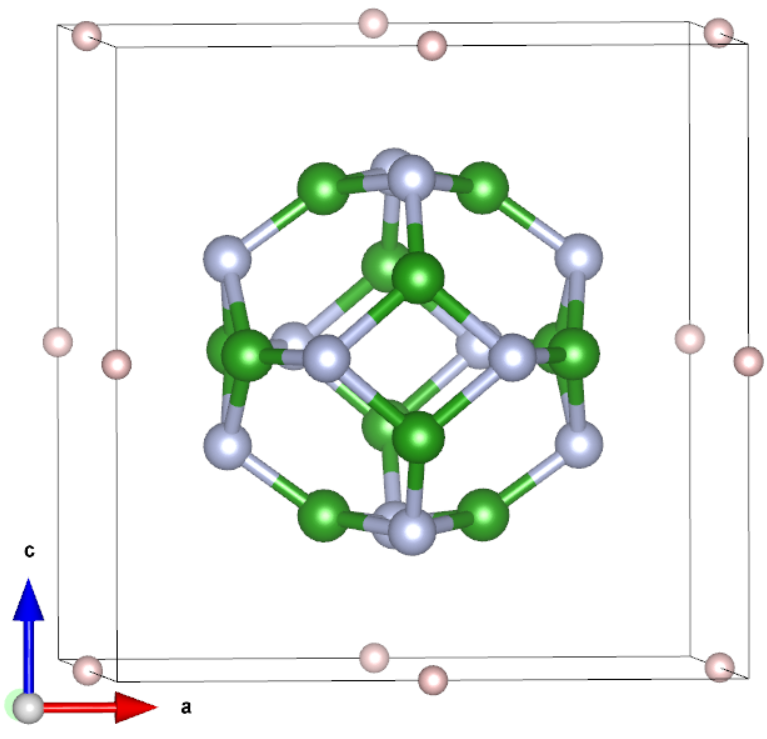**  *Pm*3-HB_4_N_4_  ***E*_f_ = -7.206 eV/atom**  a=7.663 Å, b=7.663 Å, c=7.663 Å | **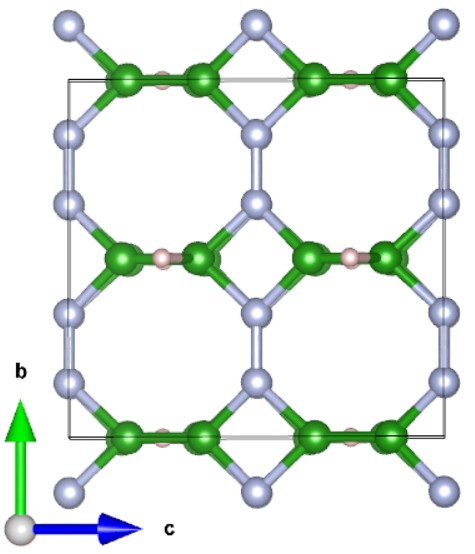** **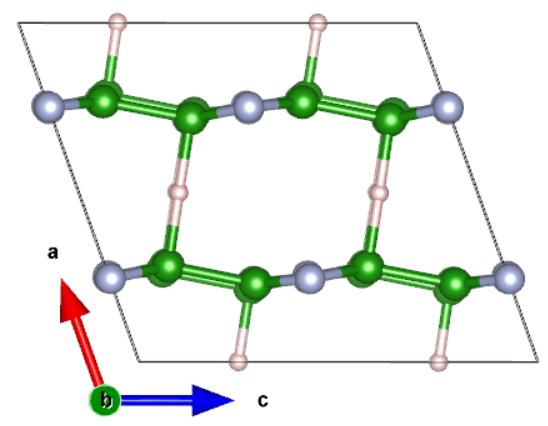**  *C*2*/m*-HB_4_N_4_  ***E*_f_ = -7.105 eV/atom**  a=6.827 Å, b=6.784 Å, c=7.571 Å |

**Fig. S3** Predicted crystal structures of HB_4_N_4_ employing CALYPSO code. The relatively stable 8 structures and their lattice parameters are presented. And the enthalpies of formation (*E*_f_) these phases were calculated.

| **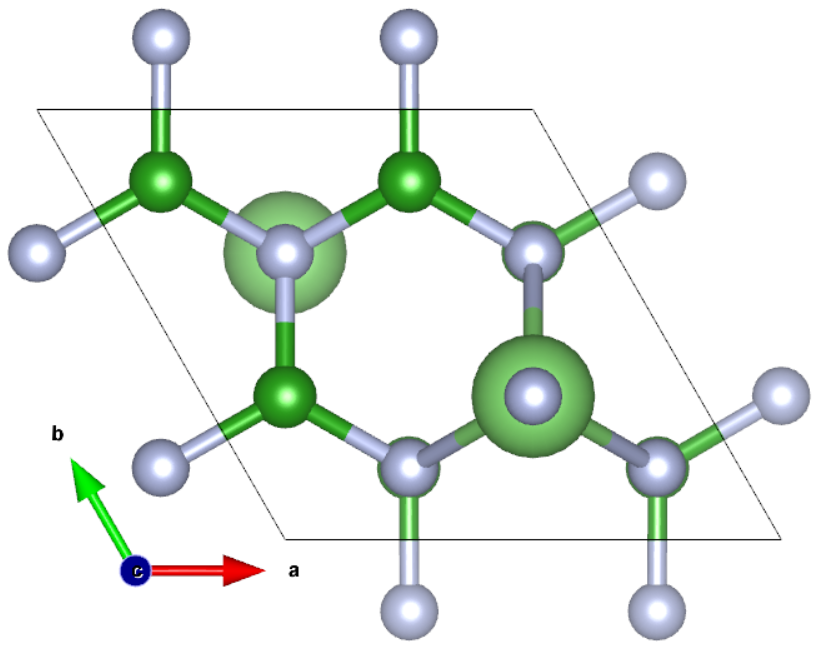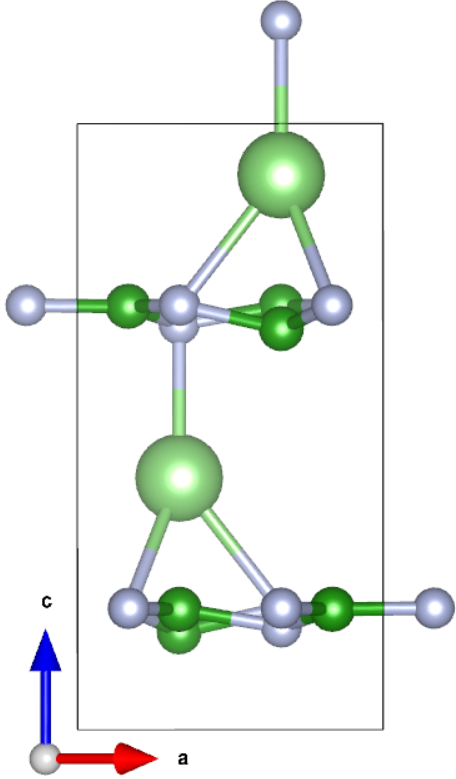**  *P*6_3_/*mmc*-LiB_4_N_4_  ***E*_f_ = -7.865 eV/atom**  a=5.022 Å, b=5.022 Å, c=8.627 Å | **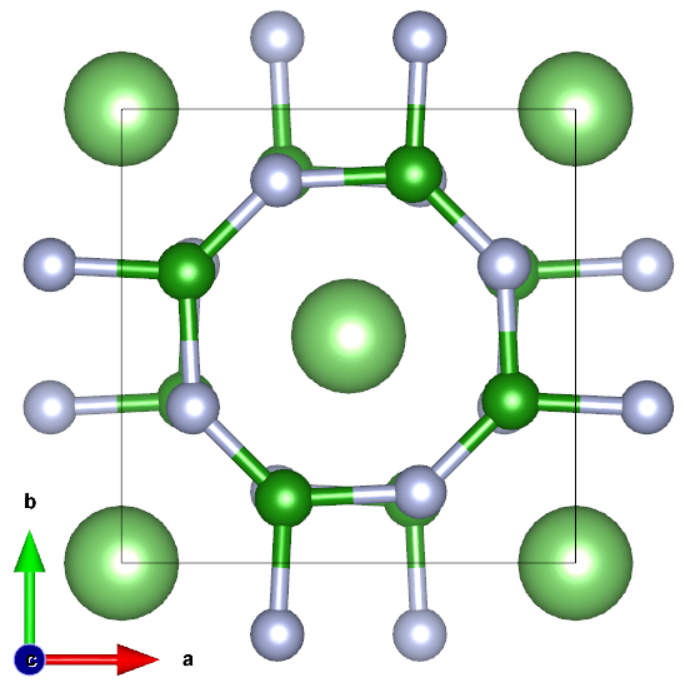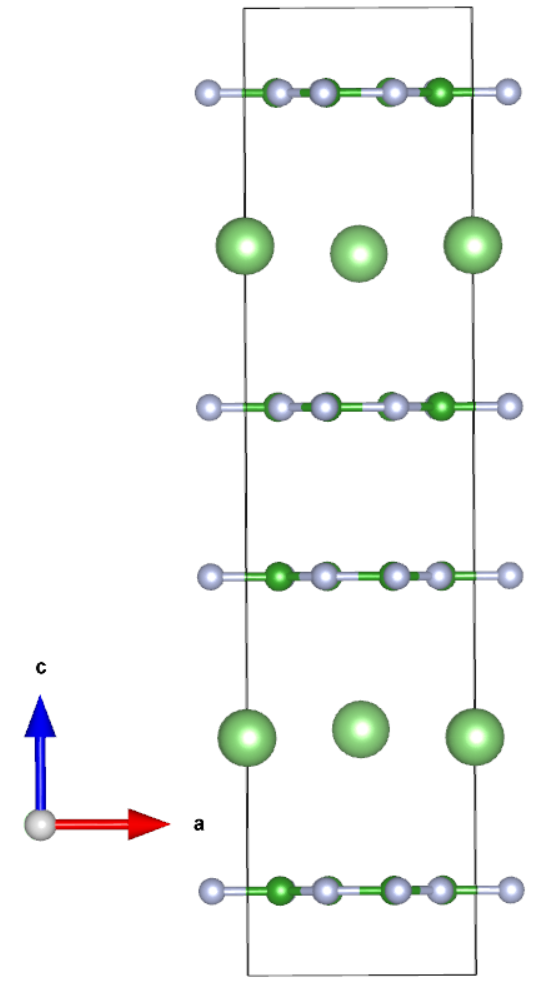**  *I*422-LiB_4_N_4_  ***E*_f_ = -7.666 eV/atom**  a=4.933 Å, b=4.933 Å, c=20.933 Å |
| --- | --- |
| **4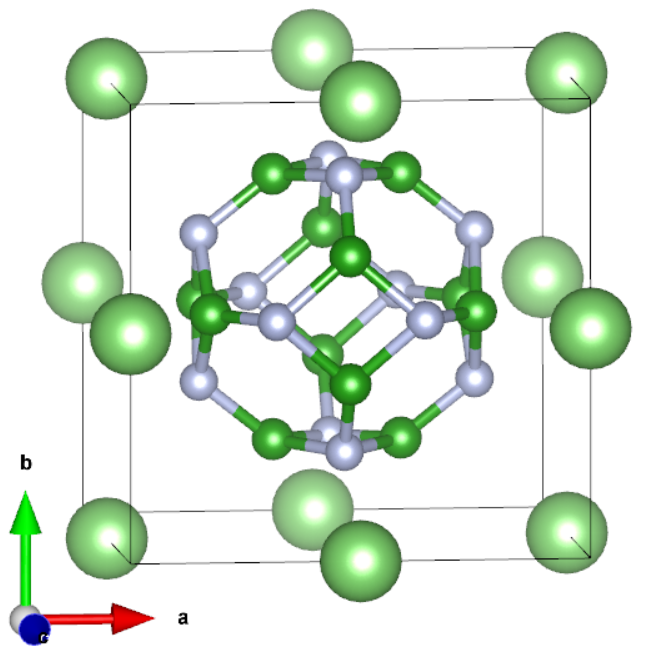**  *Pm*3-LiB_4_N_4_  ***E*_f_ = -7.314 eV/atom**  a=7.034 Å, b=7.034 Å, c=7.034 Å | **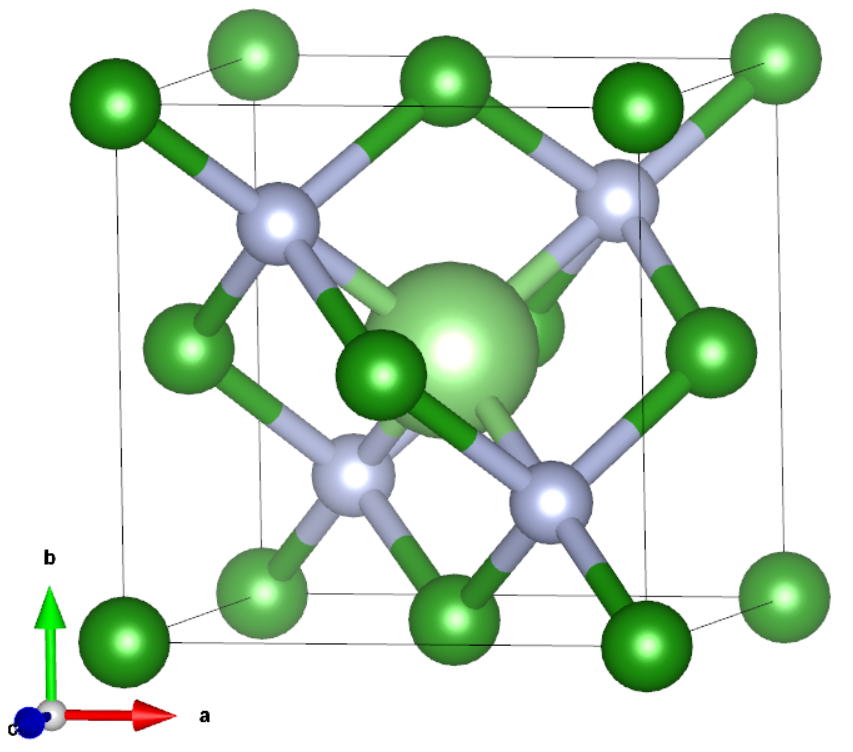**  $P\bar{4}3m$-LiB_4_N_4_  ***E*_f_ = -7.196 eV/atom**  a=3.791 Å, b=3.791 Å, c=3.791 Å |
| **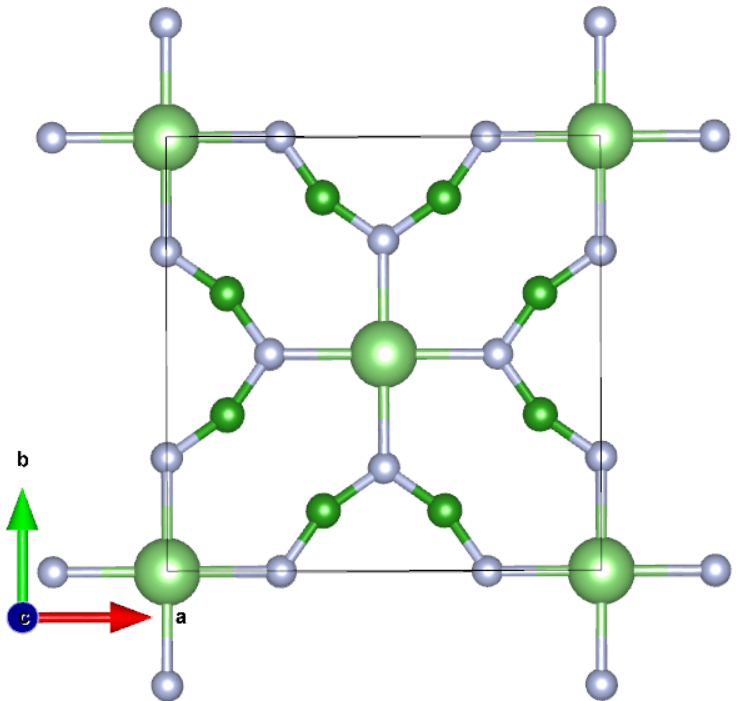** **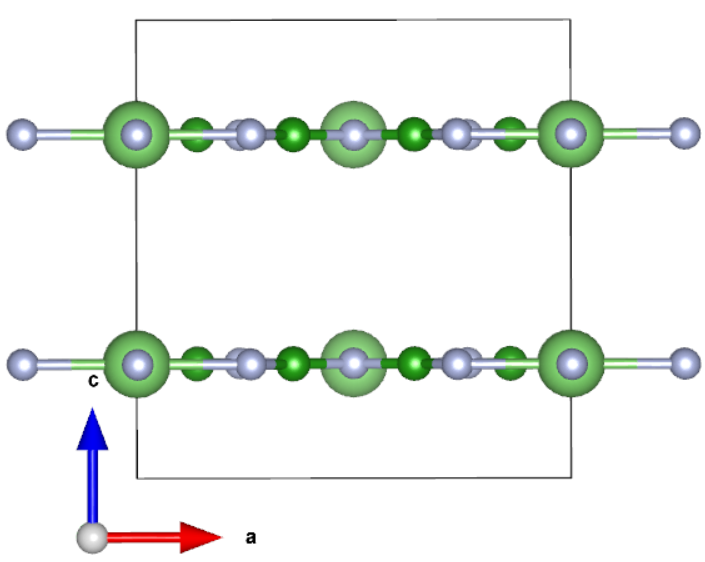**  $\bar{I4}2m$-LiB_4_N_4_  ***E*_f_ = -7.170 eV/atom**  a=8.036 Å, b=8.036 Å, c=8.477 Å | **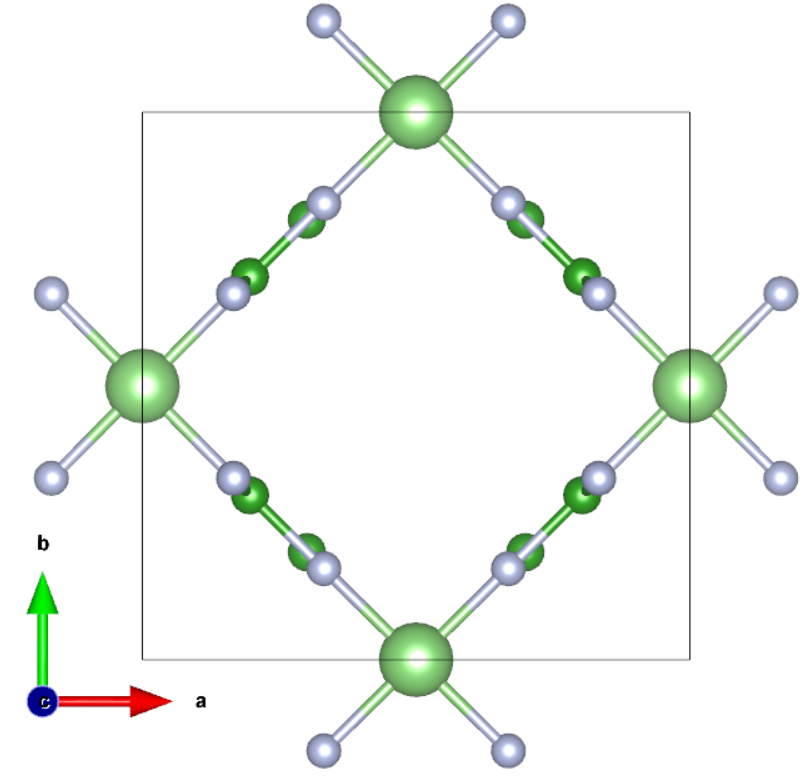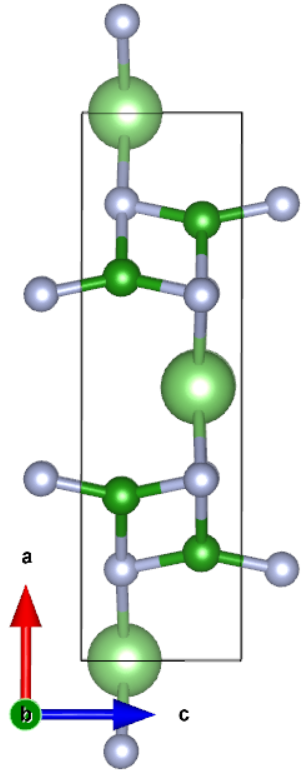**  $P\bar{4}m2$-LiB_4_N_4_  ***E*_f_ = -7.111 eV/atom**  a=9.175 Å, b=9.175 Å, c=2.685 Å |

**Fig. S4** Predicted crystal structures of LiB_4_N_4_ employing CALYPSO code. The relatively stable 6 structures and their lattice parameters are presented. And the enthalpies of formation (*E*_f_) these phases were calculated.

**4. Comparison of electronic structures between PBE and HSE methods**

To verify if the band gap is underestimated due to underestimating electron-electron exchange interactions, we have carried out the calculation of electronic structures based on the hybrid functional method (HSE)[1] with the standard parameter. Fig. S5 shows the density of states (DOS) of HB_4_B_4_ and LiB_4_N_4_, calculated by PBE and HSE functionals, respectively. The results show that PBE functional can accurately describe the electronic states of HB_4_B_4_ and LiB_4_N_4_.


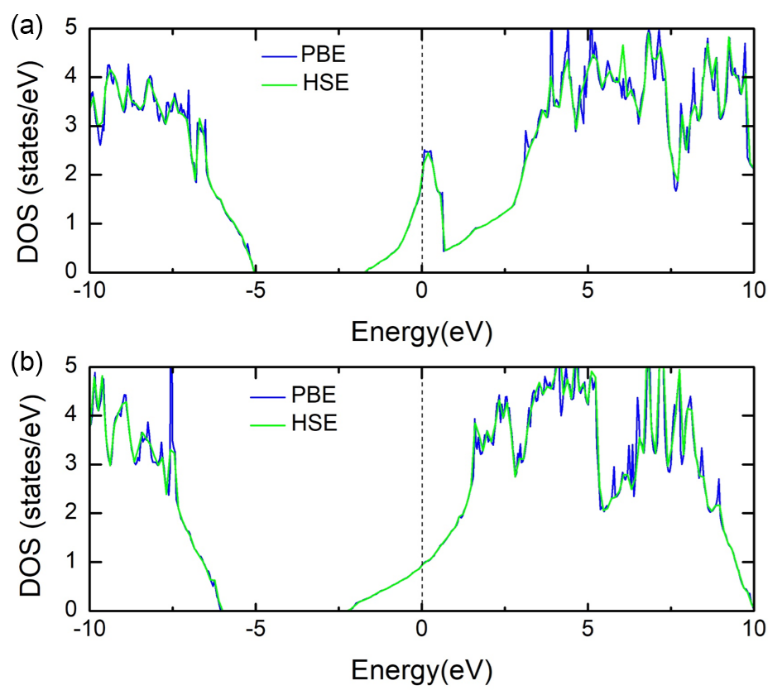


**Fig. S5** Calculated density of states (DOS) for HB_4_B_4_ and LiB_4_N_4_ by PBE and HSE functionals, respectively. (a) HB_4_B_4_, (b) LiB_4_N_4_.


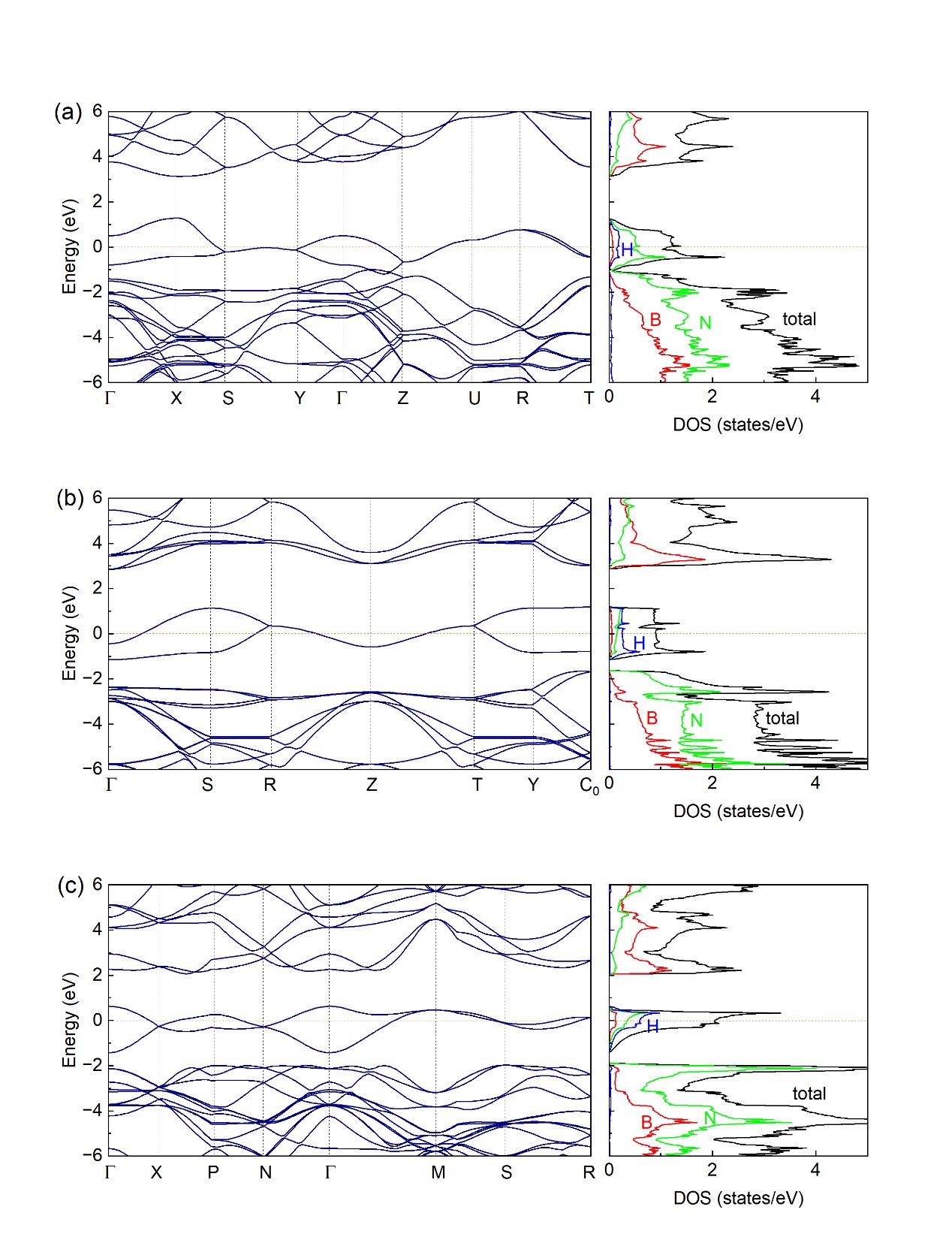


**Fig. S6** Calculated band structures and total and projected DOS of relatively stable phases. (a)-(c) are corresponding to *Pnc*2-HB_4_N_4_, *P*6_3_/*mmc*-HB_4_N_4_, and *P*4*cc*-HB_4_N_4_, respectively.


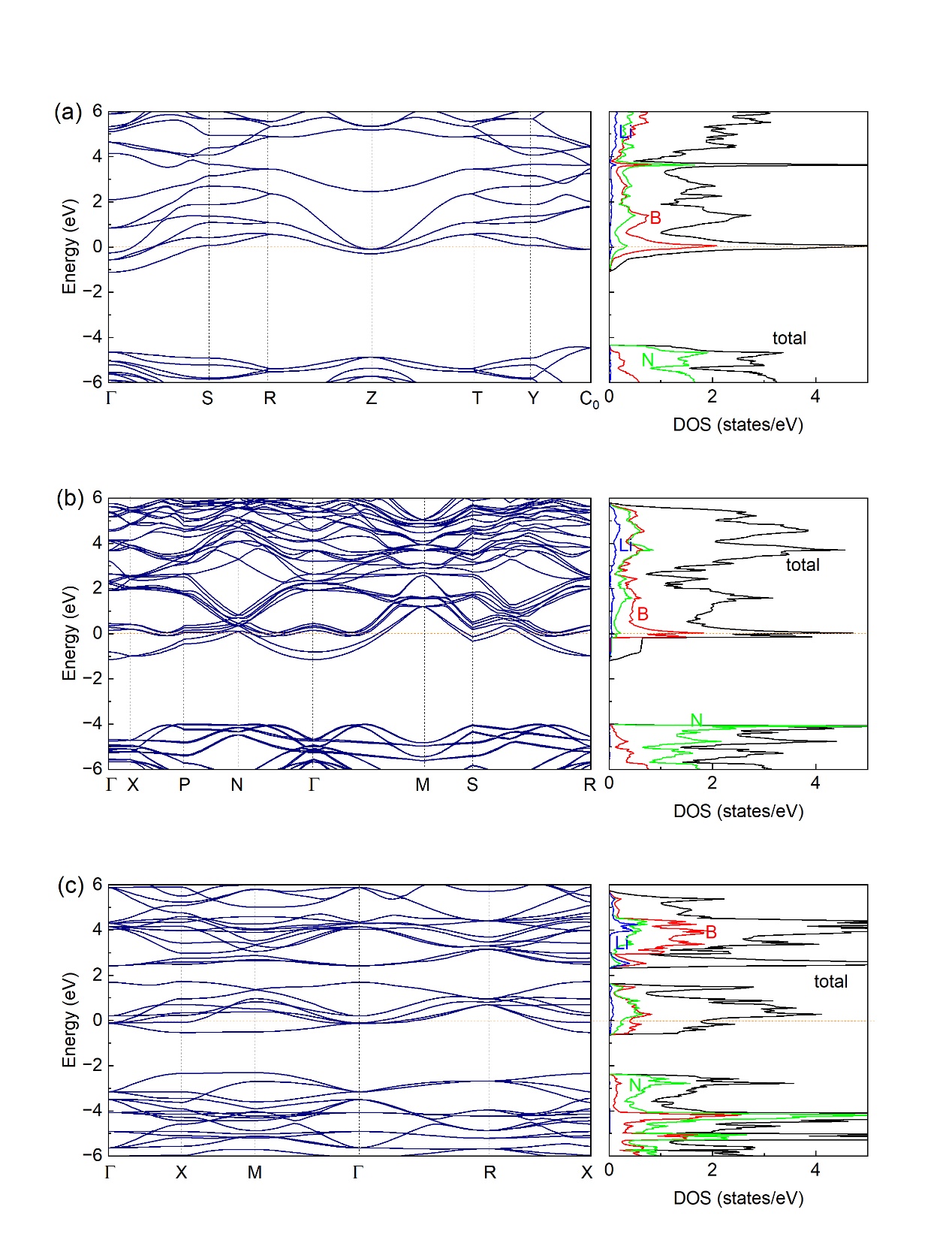


**Fig. S7** Calculated band structures and total and projected DOS of relatively stable phases. (a)-(c) are corresponding to *P*6_3_/*mmc*-LiB_4_N_4_, *I*422-LiB_4_N_4_, and *P*m3-LiB_4_N_4_, respectively.


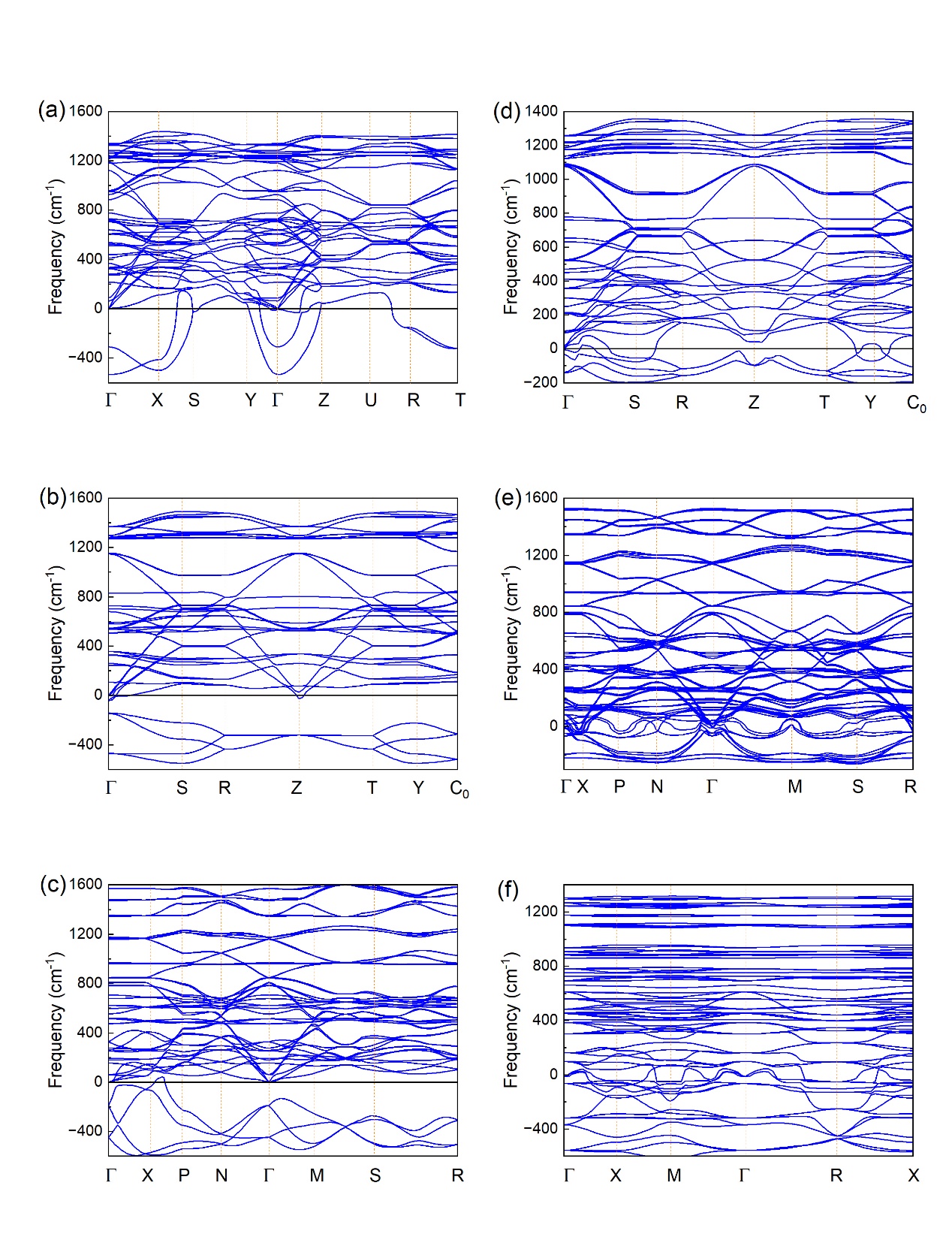


**Fig. S8** Calculated phonon spectra along high-symmetrical k-point paths of relatively stable phases. (a)-(c) are corresponding to *Pnc*2-HB_4_N_4_, *P*6_3_/*mmc*-HB_4_N_4_, and *P*4*cc*-HB_4_N_4_, respectively. (d)-(f) are corresponding to *P*6_3_*mmc*-LiB_4_N_4_, *I*422-LiB_4_N_4_, and *P*m3-LiB_4_N_4_, respectively. The imaginary frequency (Negative frequency) implies that the system is dynamically unstable at ambient pressure.


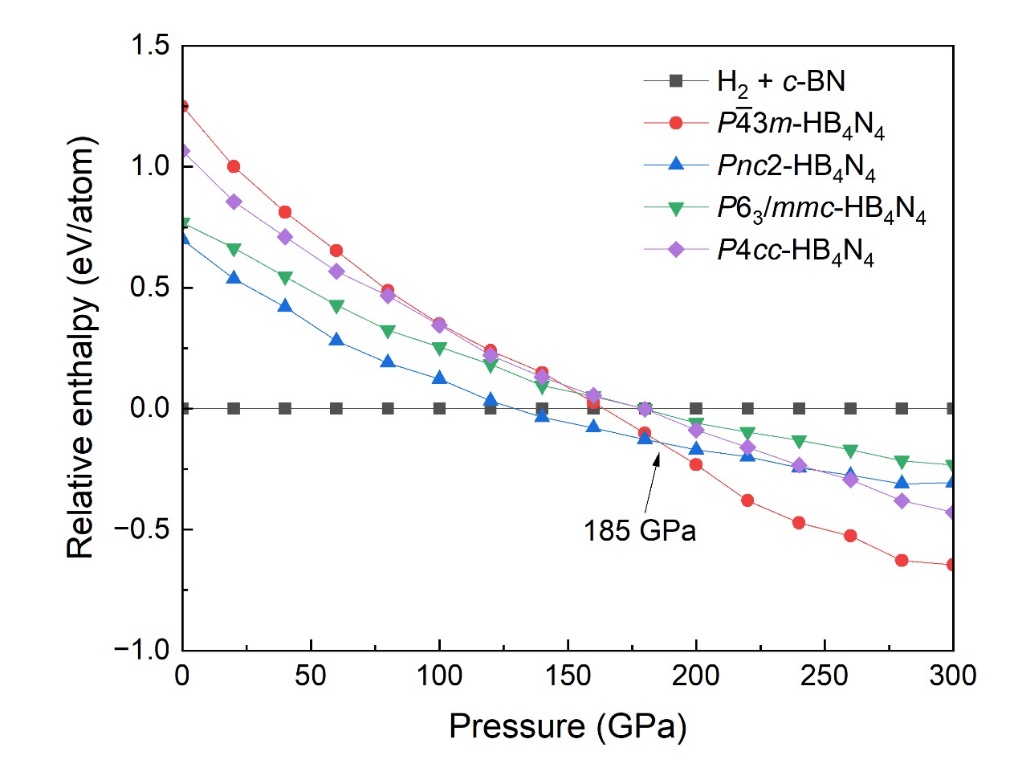


**Fig. S9** Calculate relative enthalpy as a function of pressure. The enthalpies of formation of $P\bar{4}3m$-HB_4_N_4_, *Pnc*2-HB_4_N_4_, *P*6_3_/*mmc*-HB_4_N_4_, and *P*4*cc*-HB_4_N_4_ are relative to the enthalpy of H_2_ + *c*-BN. The negative value indicates that above a certain pressure, the four phases will not decompose and can exist. Especially implying that the $P\bar{4}3m$-phase will be the most stable structure at pressures above 185 GPa.


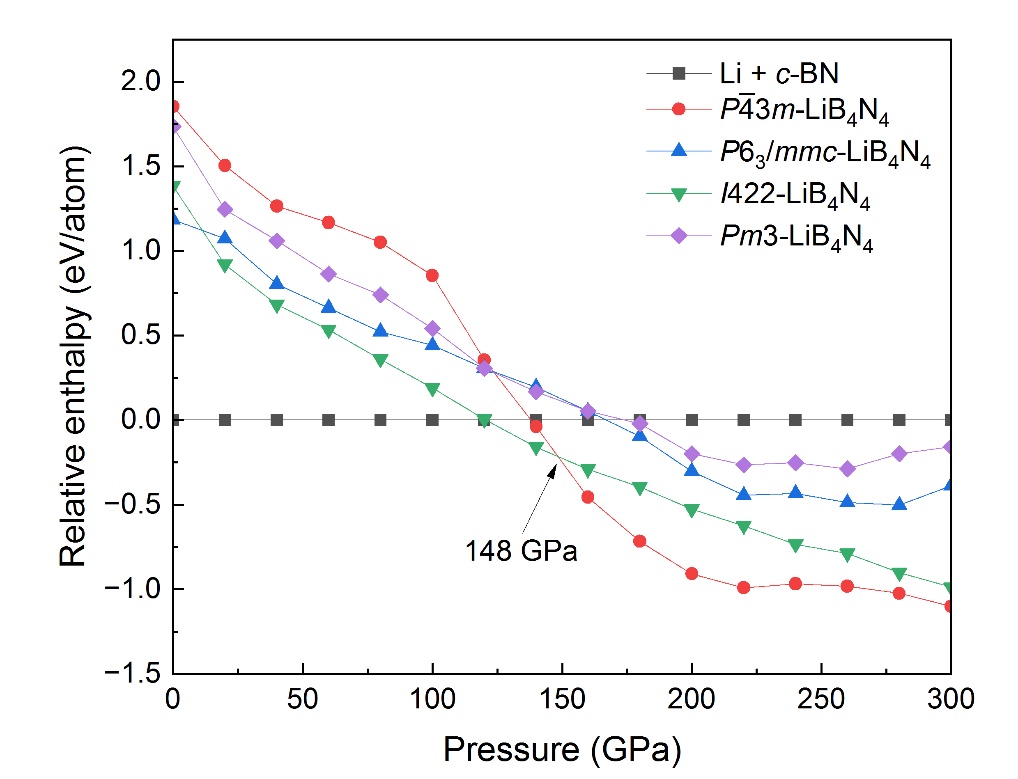


**Fig. S10** Calculate relative enthalpy as a function of pressure. The enthalpies of formation of $P\bar{4}3m$-LiB_4_N_4_, *P*6_3_/*mmc*-LiB_4_N_4_, *I*422-LiB_4_N_4_, and *Pm*3-LiB_4_N_4_ are relative to the enthalpy of Li + *c*-BN. The negative value indicates that above a certain pressure, the four phases will not decompose and can exist. Especially implying that the $P\bar{4}3m$-phase will be the most stable structure at pressures above 148 GPa.

**References:**

[1] Y. Wang, J. Lv, L. Zhu, Y. Ma, Phys. Rev. B **82**, 094116 (2010)

[2] Y. Wang, J. Lv, L. Zhu, Y. Ma, Comput. Phys. Commun. **183**, 2063-2070 (2012).

[3] J. Heyd, G. E. Scuseria, M. Ernzerhof, J. Chem. Phys. **124**, 219906 (2006).
